# Participation of Central Muscarinic Receptors on the Nervous Form of Chagas Disease in Mice Infected via Intracerebroventricular with Colombian *Trypanosoma cruzi* Strain

**DOI:** 10.3390/pathogens10020121

**Published:** 2021-01-25

**Authors:** Gabriela Maira Pereira de Assis, Micheline Freire Donato, Matheus Marques Milagre, Samantha Ribeiro Béla, Mayra Fernanda Ricci, Luara Augusta Batista, Maria Elena de Lima, Fabrício de Araujo Moreira, Rosa Maria Esteves Arantes, Marta de Lana

**Affiliations:** 1 Programa de Pós-Graduação em Ciências Biológicas (CBIOL), Núcleo de Pesquisas em Ciências Biológicas (NUPEB), Universidade Federal de Ouro Preto (UFOP), 35400-000 Ouro Preto, Brazil; gassis@aluno.fiocruz.br (G.M.P.d.A.); samantha.ribeiro@ufop.edu.br (S.R.B.); 2 Programa de Pós-Graduação em Produtos Naturais e Sintéticos Bioativos (PGPNSB), Universidade Federal da Paraíba (UFPB), 58010-340 João Pessoa, Brazil; micheline.donato@academico-ufpb.br; 3 Departamento de Ciências Farmacêuticas, Universidade Federal da Paraíba (UFPB), 58010-340 João Pessoa, Brazil; 4 Programa de Pós-Graduação em Ciências Farmacêuticas (CiPHARMA), Escola de Farmácia, Universidade Federal de Ouro Preto (UFOP), 35400-000 Ouro Preto, Brazil; matheus.milagre@aluno.ufop.edu.br; 5 Departamento de Patologia Geral, Instituto de Ciências Biológicas (ICB), Universidade Federal de Minas Gerais (UFMG), 31660-060 Belo Horizonte, Brazil; mayrafricci2014@ufmg.br (M.F.R.); rosa@icb.ufmg.br (R.M.E.A.); 6 Programa de Pós Graduação em Neurociência, Departamento de Farmacologia, Instituto de Ciências Biológicas (ICB), Universidade Federal de Minas Gerais (UFMG), 31660-060 Belo Horizonte, Brazil; luarabatista@usp.br (L.A.B.); fabriciomoreira@icb.ufmg.br (F.d.A.M.); 7 Santa Casa de Belo Horizonte: Instituto de Ensino e Pesquisa, 30150-240 Belo Horizonte, Brazil; mariaelena@faculdadesantacasabh.edu.br

**Keywords:** nervous Chagas disease, muscarinic cholinergic pathway, infection evolution

## Abstract

Acute chagasic encephalitis is a clinically severe central nervous system (CNS) manifestation. However, the knowledge of the nervous form of Chagas disease is incomplete. The role of the muscarinic acetylcholine receptor (mAChR) on mice behavior and brain lesions induced by *Trypanosoma cruzi* (Colombian strain) was herein investigated in mice treated with the mAChR agonist and antagonist (carbachol and atropine), respectively. Immunosuppressed or non-immunosuppressed mice were intracerebroventricularly (icv) or intraperitoneally (ip) infected. All groups were evaluated 15 d.p.i. (days post infection). Intraperitoneally infected animals had subpatent parasitemia. Patent parasitemia occurred only in icv infected mice. The blockade of mAChR increased the parasitemia, parasitism and lesions compared to its activation. Infected not treated (INT ip) mice did not present meningitis and encephalitis, regardless of immunosuppression. INT icv brains presented higher cellularity, discrete signs of cellular degeneration, frequent presence of parasites and focal meningitis. The immunosuppressed atropine + icv mice presented increased intracellular parasitism associated with degenerative parenchymal changes, while carbachol + icv mice presented discrete meningitis, preservation of the cortex and absence of relevant parasitism. Cholinergic receptor blockage increased impairment of coordination vs. receptor activation. Muscarinic cholinergic pathway seems to be involved in immune mediated cell invasion events while its blockade favored infection evolution, brain lesions, and behavioral alterations.

## 1. Introduction

Chagas disease (CD) or American Trypanosomiasis is a complex anthropozoonosis caused by the protozoan hemoflagellate *Trypanosoma cruzi* (*T. cruzi*), naturally transmitted to vertebrate hosts by the elimination of metacyclic trypomastigotes forms in the feces and/or urine of triatomine vectors belonging to the family Reduviidae and subfamily Triatominae [1,2]. Currently, 6–7 million people worldwide are infected with *T. cruzi*, and the disease is endemic in 21 countries in Central and South America [3]. CD represents a new global challenge due to its expansion for non-endemic countries due to the migration of infected individuals, where the parasite continues to be transmitted by mechanisms independent of the insect vector [4]. This disease presents two distinct phases, the initial acute phase that may be asymptomatic, oligosymptomatic or symptomatic with severe and fatal manifestations in children up to four years old or in immunosuppressed individuals. Patients that survive the acute phase naturally evolve for the chronic phase, which may be asymptomatic and named indeterminate clinical form, or evolve for cardiac clinical form, the more malign; digestive clinical form, mainly characterized by megaesophagus or megacolon; or mixed clinical form when cardiac and digestive manifestations are associated [5]. In any other infectious disease, the involvement of the autonomic nervous system is as important as in CD as demonstrated by the presence of lesions, denervation, and functional disorders. Denervation of the parasympathetic system has been well documented and is much more intense than denervation of the sympathetic system [6]. The destruction of the nervous system occurs mainly in the peripheral neurons, more frequently observed at the acute phase, and continually during the chronic phase (cardiac and digestive form) [7,8]. Currently, the central nervous system (CNS) impairment is recognized only at the acute phase, when meningoencephalitis presents as an important cause of death [5,6]. Most of the patients at acute nervous form are children younger than two years old, including some congenital cases [9] and immunosuppressed patients [10]) that reactivate the infection. The major macroscopic findings in the brains of patients at the acute nervous form of CD are edema, congestion, and scattered petechial hemorrhages. The main histopathological finding is encephalitis in multiple foci in the CNS, characterized by a nodal arrangement of microglia, macrophages, neutrophils, and astrocytes and mild perivascular lymphocytic infiltrate [11]. Amastigotes are present, in nests or cellular processes in astrocytes or, less frequently, in the center of inflammatory nodules [12]. Changes in nerve cells, when present, are not specific and degenerative, affecting only cells located near or inside inflammatory foci. Clinical manifestations are usually mild, occasionally with torpor or excitation, and in a few cases are severe, with seizures and focal neurological signs [13]. The usual inflammatory changes and the high frequency of trypomastigote forms in the cerebrospinal fluid is explained by the presence of inflammation and parasites in the leptomeninges [14]. In most patients with the symptomatic acute nervous form, the lesions, parasitism, and all manifestations, including neurological signs and symptoms, disappear spontaneously without apparent sequelae [15], what reinforces the view against the existence of a chronic nervous form of CD [9,15,16]. These observations lead to be ignored the nervous form of the CD. Neurological and behavioral changes were also observed in animals, such as paralysis of the hind limbs, seizures, difficulty in standing and depression detected in behavioral tests [17,18]. Chagasic encephalitis is invariably associated with chagasic myocarditis. This association is responsible for the high mortality rate and the severity of the acute phase of the disease [19]. Currently, peripheral nervous system (PNS) alterations are better understood, and dysautonomia secondary to ganglia and nerve endings of the sympathetic and parasympathetic autonomic nervous system have been consistently implicated in the pathophysiology of cardiomyopathy and chagasic megasyndromes [8,20].

Two fundamental theories on the genesis of chagasic cardiomyopathy are under consideration. The neurogenic theory predicts consequences of the parasympathetic deprivation on postganglionic vagal neurons during the acute phase of infection, and promote trophic changes such as in the regulation of the muscarinic cholinergic receptor population, like up-regulation in the myocardium. However, according to the immunogenic theory, a putative agonist activity of autoantibodies induces subsensitivity and desensitization of the muscarinic acetylcholine receptors (mAChR) [21,22,23,24], which suggest changes in the peripheral cholinergic system function. However, the bias between central cholinergic pathways and the CNS damage needs clarification. The mAChRs are metabotropic receptors signalized through G-proteins. The mammalian mAChR subtypes (M1–M5) are five and expressed in most tissues and cell types where exert diverse physiological actions dependent on the cellular location and identity of receptor subtypes [25]. Some studies have reported the involvement of cardiac cholinergic functions in rats with chronic CD [26]). Other study demonstrated the presence of autoantibodies against the second extracellular loop of the human heart mAChR (M2 type) in patients with CD [27]). The functional relevance of cholinergic receptors in various peripheral and central functions relies on scarce anatomopathological and physiological knowledge of the mechanisms of nervous manifestations in CD. The neuronal infection by the parasite and its consequences to the host as well as the elucidation of the pharmacological pathways involved in *T. cruzi* infection in CNS demand further investigation.

Thus, this study aimed to investigate the participation of the muscarinic cholinergic pathway in the process of acute infection by *T. cruzi* using immunosuppressed and non-immunosuppressed mice, infected via intracerebroventricular (icv), and previously treated with the muscarinic cholinergic receptor agonist, carbachol and the antagonist, atropine.

## 2. Results

### 2.1. Curve of Parasitemia and Mortality

#### 2.1.1. In Immunosuppressed Animals

**Figure 1:**
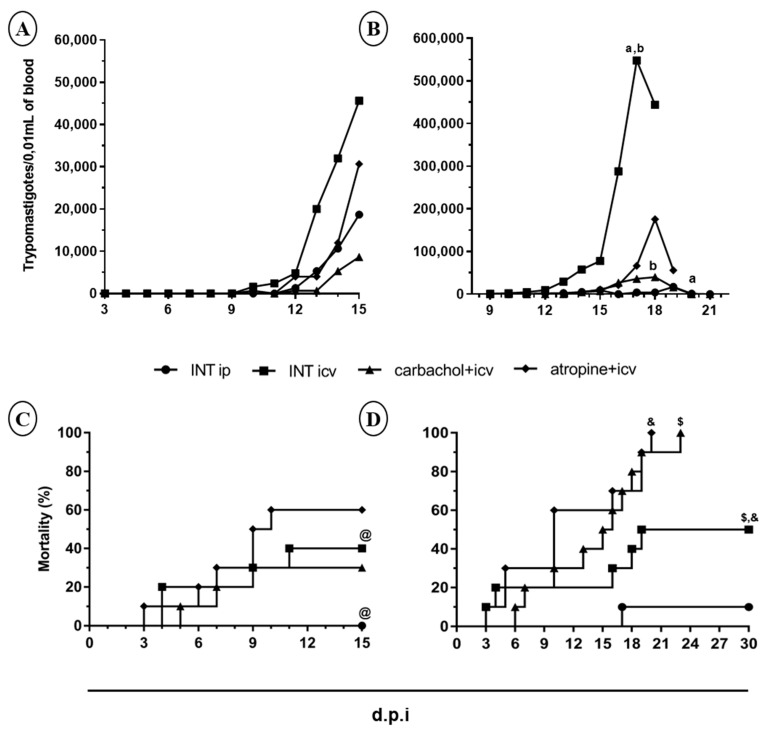
Curve of mean parasitemia (**A**,**B**) and mortality (**C**,**D**) in Swiss mice infected via intracerebroventricular (icv) and intraperitoneal (ip) with 2000 trypomastigotes of Colombian *Trypanosoma cruzi* strain, immunosuppressed treated with carbachol (agonist cholinergic) or atropine (antagonist cholinergic) of the muscarinic receptors and evaluated 15 (**A**,**C**) and 30 (**B**,**D**) days post-infection. INT icv: infected not treated via icv; INT ip: infected not treated via ip; carbachol + icv: previously treated via icv with carbachol and infected via icv; atropine + icv: previously treated via icv with atropine and infected via icv. Statistical analyses by Kruskal-Wallis test and pos-test Dunn’s (**A**,**B**) and Survival curve and Log-rank (Mantel-Cox) Test (**C**,**D**) (GraphPad Prism 7). a,b Different letters indicate statistical significant difference between the mean parasitemia (*p* < 0.05). ^@,$,&^ Different symbols indicate significant statistical difference of mortality (*p* < 0.05).

No statistical differences were observed in parasitemia among all groups when evaluated up 15 d.p.i. (Figure 1A), but the rate of mortality of the animals of the INT icv (40%) was significantly higher (*p* < 0.05) than INT ip group (0%) (Figure 1C and Table 1). In animals evaluated up 30 d.p.i. the parasitemia was always higher than the observed up 15 d.p.i., but statistical differences were observed only between INT icv and INT ip groups (*p* < 0.05) (Figure 1B). Interestingly, in animals evaluated up to 30 d.p.i. the rate of mortality of the INT icv group of 50% was also higher than INT ip group (10%). All animals of the carbachol + icv and atropine + icv groups died before 30 days (20 to 23 d.p.i.). These rates of mortality (100%) were different from the control groups (INT ip and INT icv), and more precocious in the atropine + icv group (Figure 1D and Table 1).

#### 2.1.2. In Non Immunosuppressed Animals

Curve of parasitemia was not obtained in mice non immunosuppressed evaluated up to 15 d.p.i. and 30 d.p.i. Parasites were observed in only one animal of the INT icv group evaluated up 30th d.p.i. and in two mice of the INT ip group only one day, at 16th and 19th d.p.i (data not shown). Mortality rates in non-immunosuppressed mice were low (0% to 20%) (Table 2) and similar in all experimental groups when compared to immunosuppressed mice (Table 1).

Interestingly, at 30 d.p.i. all animals of the control groups (INT icv and INT ip) were 100% positive in HC and PCR and the mortality was low with rates of 10% and 50%, respectively, while 100% of mortality occurred in the carbachol + icv and atropine + icv groups (Figure 1D and Table 1), what makes these evaluations impossible. Sera from all surviving and immunosuppressed animals were not reactive with *T. cruzi* antigens as expected (Table 1).

#### 2.1.3. In Non Immunosuppressed Animals

Non-immunosuppressed animals presented lower rates of positive parasitological tests (HC and PCR) than animals of the immunosuppressed groups in both evaluations (15 and 30 d.p.i.). The only exception was the atropine + icv group evaluated 30 d.p.i. that presented 100% of positivity in both parasitological tests and different from the other experimental groups (Table 2). The positive rates were also higher in surviving animals evaluated later (30 d.p.i.). The mortality was similar in all groups (Table 2) and lower (0 to 20%) than the observed in immunosuppressed animals (90–100%) (Table 1). ELISA test was reactive in all animals (Table 2).

### 2.2. Histopathological Data

Descriptive and semi-quantitative analyses of brain samples summarized the histopathological findings and highlighted the comparisons between the groups.

#### 2.2.1. Control Groups Immunosuppressed and Non Immunosuppressed

**Figure 2:**
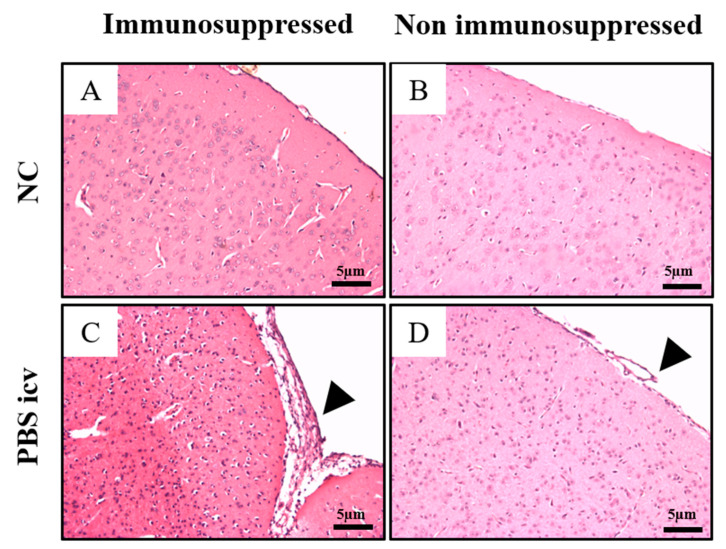
Brain histopathological aspects of Swiss mice immunosuppressed (**A**,**C**) and non immunosuppressed (**B**,**D**) control groups. NC: normal control and PBS icv: injected with Phosphate Buffer Solution via icv evaluated at 15 days. (**A**–**D**) objective magnification of the 10×. Arrowheads indicate reactive changes of meninges.

**Figure 3:**
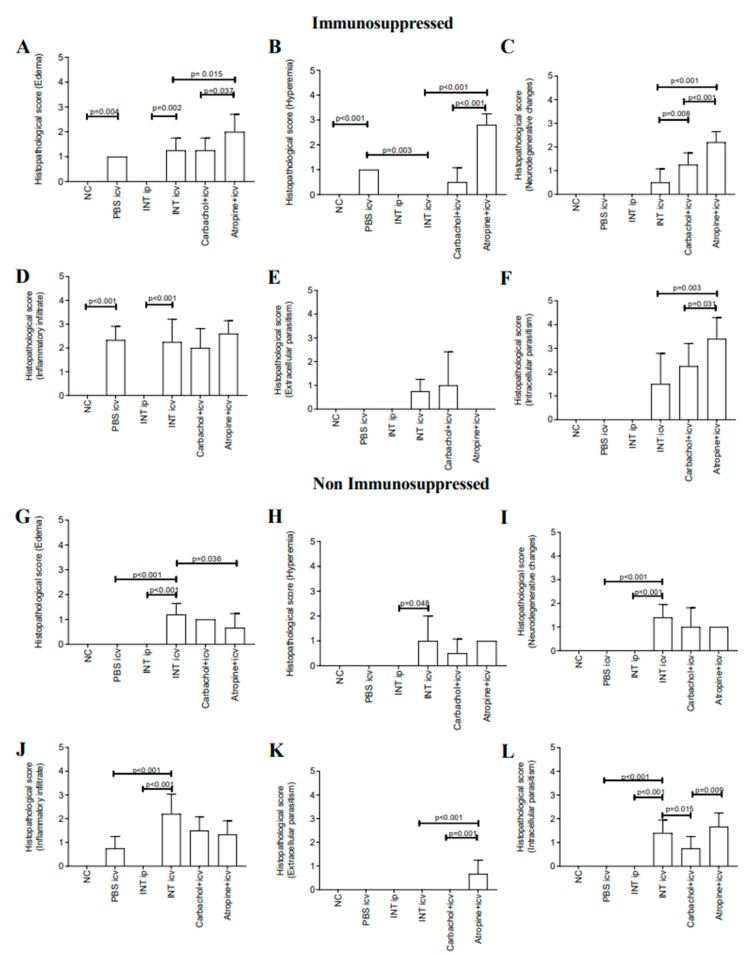
Semi-quantitative histopathological score in brain of Swiss mice immunosuppressed (**A**–**F**) and non immu-nosuppressed (**G**–**K**) evaluated 15 days post infection. Edema (**A**,**G**), Hyperemia (**B**,**H**), Neurodegenerative changes (**C**,**I**), Inflammatory infiltrate (**D**,**J**), Extracellular parasitism (**E**,**K**), Intracellular Parasitism (**F**,**L**); Experimental groups: icv: Group infected via icv, carbachol + icv: Group previously treated via icv with carbachol (3 μM) and infected via icv; atropine + icv: Group previously treated via icv with atropine (20 μM) and infected via icv; Control groups: INT ip: Group infected not treated via ip; INT icv: Group infected not treated via icv; PBS icv: Group inoculated via icv with 5 μL of Phosphate Buffer solution (PBS); NC: Normal control. Statistical analysis by ANOVA One-way, with SNK post-hoc (Prisma Graphpad version 7.0).

At day 15th no significant changes were present in normal control (NC) and INT ip groups immunosuppressed (Figure 2 and Figure 3A–F). Only discrete changes were found in non-immunosuppressed groups (Figure 3C,D,G–L). There are focal intracortical and hypothalamic areas with increased cellularity and slight signs of cellular degeneration, in addition to presence of parasite nests.

#### 2.2.2. Immunosuppressed Animals Treated with Carbachol and Atropine (15th d.p.i.)

**Figure 4:**
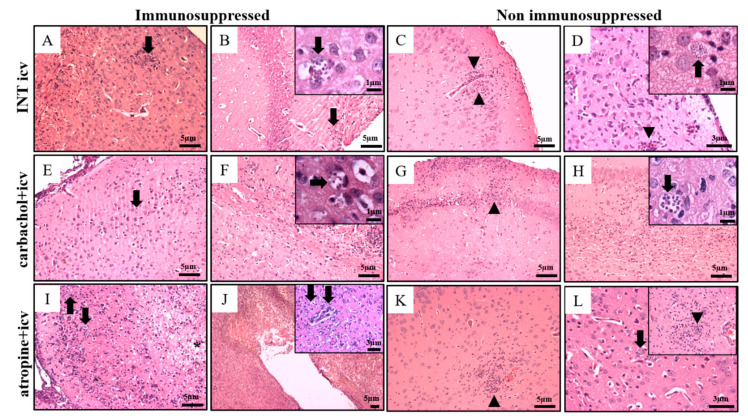
Brain histopathological aspect of Swiss mice infected with Colombian *Trypanosoma cruzi* strain immunosuppressed and non immunosuppressed and evaluated 15 days post-infection. INT icv: infected and not treated via icv immunosuppressed (**A**,**B** and detail) and non immunosuppressed (**C**,**D** and detail). carbachol + icv: previously treated with carbachol via icv infected and immunosuppressed (**E**,**F** and detail) and non immunosuppressed (**G**,**H** and detail). atropine + icv: previously treated with atropine via icv infected and immunosuppressed (**I**,**J** and detail) and non immunosuppressed (**K**,**L** and detail). (**A**–**C**,**E**–**H**,**K**): 10× objective; (**J**): 4× objective; (**D**,**L**): 20× objective. (**B**,**D**,**F**,**H**) and details: 100× objective; (**J**,**L**) detail: 20× objective. Note: Arrows indicate nest of parasites; Arrowheads indicate inflammatory infiltrate.

Except for extracellular parasitism, the atropine + icv animals presented higher score for all evaluated lesion parameters (Figure 3A–F). In the carbachol + icv groups, immunosuppressed and non-immunosuppressed, the meningitis was discrete. In the presence of cholinergic receptors activation (carbachol + icv) the cortex presented good preservation of anatomical and cellular structures of the brain (Figure 4E).

However, degenerative changes of the white matter and corpus callosum, in association with inflammatory changes, in the transition cortex-hippocampus, callous body, and internal capsule were observed (Figure 4F, and detail; Table 3). Except for extracellular parasitism and inflammatory infiltrate (Figure 3D,E), all other lesion parameters scored significantly higher in the atropine + icv group compared to INT icv. The atropine + icv group (Figure 4I,J) presented higher scores for edema, hyperemia, neurodegenerative changes and intracellular parasitism compared to the carbacol + icv group (Figure 3E,F and Figure 4A–F). In the group of animals that had the muscarinic cholinergic receptors blocked (atropine + icv) all areas of the brain were parasitized. Important degenerative changes of the parenchyma, such as necrosis in the periventricular area, cortical degeneration in addition to edema were observed (Figure 4I,J and detail; Table 3). There was also important parasitism in the parenchyma and meninges with the presence of intact amastigotes nests. In several areas, the parasites and inflammatory infiltrate were in close association with neuronal degenerative changes and cell death. Extensive damage in the hippocampus and cortex was observed. The parasitism and parenchymal vascular reactivity were more intense in animals of the atropine + icv group than control INT icv group. All animals immunosuppressed died before 30 d.p.i. (Table 1) and consequently were not histopathologically evaluated.

#### 2.2.3. Non Immunosuppressed Animals Treated with Carbachol and Atropine (15th and 30th d.p.i.)

**Figure 5:**
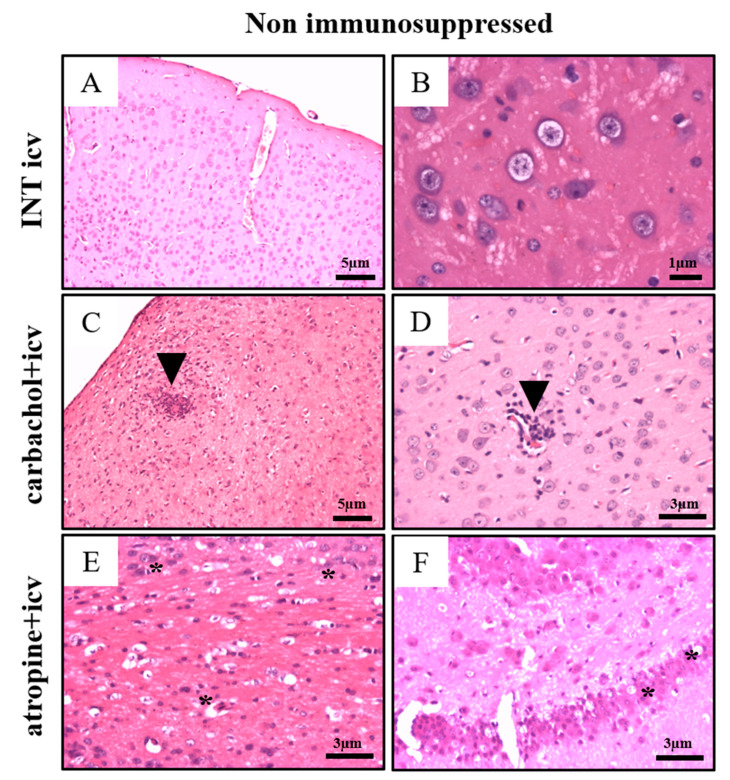
Histopathological aspects of brain of the Swiss mice infected with Colombian *Trypanosoma cruzi* strain non immunosuppressed evaluated at 30 days post-infection. INT icv: infected not treated via icv (**A**,**B**). carbachol + icv: previously treated via icv with carbachol and infected via icv (**C**,**D**), atropine + icv: previously treated via icv with atropine and infected via icv (**E**,**F**). (**A**,**C**): objective magnification of 10×. (**D**–**F**): objective magnification of 20×. (**B**): objective magnification of 40×. Asterisk (*): signs of neurodegeneration; Arrowhead: Inflammatory infiltrate.

In the INT icv group evaluated at 15 d.p.i. a focal inflammatory infiltrate was observed, associated with areas of neuronal degeneration, cellular necrosis, and vacuolization of the white matter (Figure 4C,D and detail; Table 3). The meninges also presented intense mononuclear inflammatory cells. INT icv animals presented significant edema that decreased when treated with atropine (Figure 3G), and intracellular parasitism that decreased with carbachol treatment (Figure 3L). In the cortex and hypothalamus, the inflammatory lesions were focal and extracellular parasitism was not observed (Figure 3G,H and Figure 4; Table 3). The only non-immunosuppressed group that presented extracellular parasites was the atropine + icv (Figure 3K) that also increased the intracellular parasitism compared to the carbachol + icv group (Figure 3L). In carbachol + icv group, evaluated at 15th d.p.i, the infection was focal and the degenerative changes were directly associated with the inflammatory process (Figure 4G,H and detail; Table 3), while in atropine + icv group evaluated at 15th d.p.i., focal meningitis and diffuse inflammatory infiltrate in the cortical region, intense vascular reactivity, gliosis, presence of necrotic cells, and some isolated parasites and amastigote nests were observed (Figure 4K,L and detail; Table 3). At 30th d.p.i. atropine + icv group presented focal mononuclear inflammation, predominantly perivascular in the cortico-thalamic transition region, without presence of parasitism and with few degenerative changes (Figure 5). The parasitism and alterative changes occurred evenly through the cortex, hypothalamus, and cortico-thalamic transition.

Although the persistence of vascular reactivity profile had been observed, only rare inflammatory foci, without meningitis were found. There was also the presence of parenchymal vacuolization in the corpus callosum and hippocampus (Figure 5E,F). No significant changes or parasites were observed (Figure 1A,B). Notice that the immunosuppressed animals showed more intense meningitis at 15 d.p.i (Figure 4C,D; Table 3).

### 2.3. Behavioral Data—Motor and Equilibrium Evaluation (Rotarod Test) 

#### 2.3.1. Immunosuppressed Animals Treated with Carbachol and Atropine

**Figure 6:**
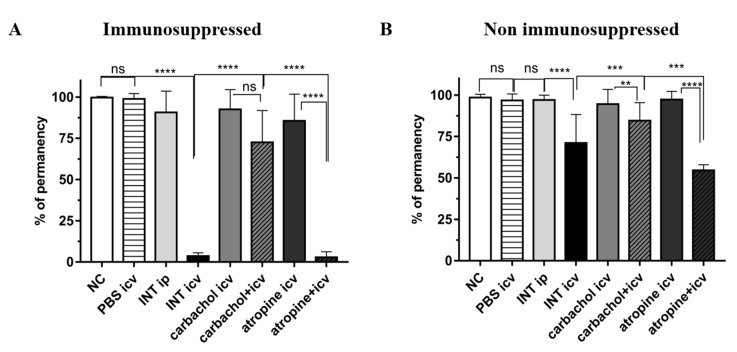
Percentage of time reduction permanency on the rotating stem of mice infected with Colombian *Trypanosoma cruzi* strain immunosuppressed (**A**) and non immunosuppressed (**B**), submitted to the Rotarod behavioral test. Test groups: INT icv: infected not treated via icv; INT ip: infected not treated via ip; carbachol + icv group: previously treated via icv with carbachol (3 μM) and infected via icv; atropine + icv group: previously treated via icv with atropine (20 μM) and infected via icv. Control groups: NC: Normal control, PBS icv: injected with Phosphate Buffer solution (PBS) via icv; carbachol icv: treated with carbachol (3 μM) via icv; atropine icv: treated with atropine (20 μM) via icv. Statistical analysis by ANOVA One-way, with Newman-Keuls post-test (Prisma Graphpad version 7.0). ns: Not significant difference; Statistical difference: < 0.05; ** *p* < 0.01; *** *p* < 0.001; **** *p* < 0.0001.

The comparison between the group of mice with the cholinergic receptors activated (carbachol + icv) and blocked (atropine + icv) showed that 96% of reduction in the time of permanency of the animals in the rotarod system was observed in the last group. This rate of reduction was different from the control group atropine icv (*p* < 0.0001). However, no differences in the time spent on the rotating system were observed between the carbachol + icv group and its control (carbachol icv and not infected) (Figure 6A).

Motor coordination was not impaired in animals of carbachol + icv group compared to group INT icv, which remained approximately 20 times more (*p* < 0.0001) in the Rotarod apparatus. The INT icv group remained 20 times less on the rotating system than the animals INT ip, NC, and PBS icv (*p* < 0.0001). The control groups NC and PBS icv remained on the rotating system for all time of the test without movement and balance disorders.

#### 2.3.2. Non Immunosuppressed Animals Treated with Carbachol and Atropine

No presence of any impairment of motor coordination was observed in the carbachol + icv group when compared with animals of atropine + icv and INT icv (*p* < 0.001) groups. The time spent in the Rotarod apparatus of animals of the atropine + icv group in the system was also 45% less than the control groups atropine, NC and PBS icv (*p* < 0.0001) (Figure 6B).

## 3. Discussion

We investigated the neuropathological and behavioral changes induced by infection with the Colombian strain of *T. cruzi* and immunosuppression in mice inoculated via icv. The icv route was chosen to guarantee the infection of the nervous tissue and allow studying correlations between histopathology (Figure 2) and pharmacological mechanisms and the CNS clinical manifestations in murine model. We also compared this route of inoculation with the classically used ip route, also artificial.

In not immunosuppressed animals, the parasitemia was absent, and when occurred it was late and lasting for one day in only three/forty animals, being one of the INT icv group and two mice of the INT ip group. The absence of parasitemia may have occurred due the low inoculum of 2000 infective forms. In addition, the trypomastigotes derived from cell culture are less infective than blood trypomastigotes [28]. Moreover, Colombian strain has a predominance of broad trypomastigotes forms that interact slowly with the host cell than the slender ones. They also present a long pre-patent period (around 10–14 days), even in Swiss mice infected via ip [29]).

Severe acute phase clinical forms of human Chagas’ disease with cardiomyopathy and meningoencephalitis are rare and depend on the parasitemia of the *T. cruzi* strain involved. We induced immunosuppression as a strategy for achieve high parasitemia sufficient and able to cause nervous lesions and for verify its correlation with histopathological and clinical changes, similarly to Da Mata’s approach [12]. The higher parasitemia in immunosuppressed animals compared to non-immunosuppressed ones was observed both, at 15 and 30 d.p.i. (Figure 1A,B), being higher at the second evaluation, confirming that the occurrence of patent parasitemia in animals infected with the Colombian strain is late as demonstrated [29]. The parasitemia increased until the 17th or 18th d.p.i., indicating that the immunosuppression favored the parasite’s multiplication, mainly in the INT icv. The INT ip group presented the lowest parasitemia among all groups. Possibly, the icv route favored the parasitemia, maybe because the parasites were protected from the macrophages more present in the peritoneal cavity. Macrophages are the first defensive cells to be infected, which also rapidly trigger the immune response against the parasite and control the parasitemia. Moreover, in the CNS, an immunologically privileged site, parasites are relatively protected against the general host immune response that start since the first week after infection, even though blood-born macrophages and microglia role in brain infections have been discussed in rats [30]. Animals of the carbachol + icv group immunosuppressed presented lower parasitemia than atropine + icv, suggesting that the infection was favored by muscarinic receptors blocking.

Pioneer studies in vitro of *T. cruzi*-myoblast cell interaction showed that following the onset of contact and stability, Ca^2+^ and cell phosphorylated intermediates were required for the subsequent phase of maximum parasite membrane-myoblast interaction [31]. The hypothesis proposed for the mechanism of adhesion of *T. cruzi* attachment molecules to extracellular loops of pairs of mAChR and β-adrenergic receptors of type I host muscle cell sarcolemma [32] is still not clear. Growing evidence suggests that *T. cruzi* uses the tyrosine receptor kinase TrkA (tropomyosin-related kinase A) to invade neural cells [33] and TrkC in neural and nonneural cells like Schwann cells and astrocytes showing that TrkC mediated cell entry is important for a proper *T. cruzi* infection in vivo. *T. cruzi*, through its trans-sialidase (parasite-derived neurotrophic factor or PDNF located on the *T. cruzi* trypomastigote surface through a glycosylphosphatidylinositol (GPI) anchor) mimics naturally Trk neurotrophin ligands in mammalian hosts and then triggers the activation of these receptors allowing the parasitism.

We hypothesize that the non-specific cholinergic drugs injected 30 min before the *T. cruzi* infection had induced changes in the dynamic of intracellular Ca^2+^ and phosphorylated proteins, due mAChR activation. Cholinergic drugs probably promoted an internalization or desensitization of mAChR [34], reducing the parasitemia and hindering the parasitism performance of *T. cruzi* in brain tissues.

The mortality throughout the infection was higher in immunosuppressed than in non-immunosuppressed animals, mainly in the groups treated with antagonist (atropine + icv) versus the agonist (carbachol + icv) of the mAChR, followed by the group icv infected and ip infected, respectively (Figure 1C,D). In association with the high parasitemia, the immunosuppression favored the tissue parasitism and lead to higher mice mortality at 30th d.p.i. than at 15 d.p.i. High parasite multiplication may also have caused damages in more areas or vital organs of the host, leading to the higher mortality observed in animals infected treated with cholinergic drugs and immunosuppressed. In the mammalian nervous system, mAChR action is primarily modulators of synaptic transmission, cognitive regulation, sensory, motor and autonomic functions, and implicated in the pathophysiology of neurological and psychiatric illnesses [35]. Moreover, cyclophosphamide facilitates the development of autoimmune disease due to a selective depletion of the humoral and cellular immunity. Although the immunosuppression here used was less intense, in one study with Sprague Dawley rats, infected with *T. cruzi* and treated with cyclophosphamide (20 mg/kg) twice a week, in five successive doses and evaluated six months later, similar results were observed. The cardiac function using selective M1, M2, M3, and M4 muscarinic antagonists facilitated the development of the chronic chagasic cardiomyopathy attributed to the promotion of parasympathetic cardiac disturbances that appeared due to alterations on the mAChR [36]. Thus, an unbalance of the muscarinic cholinergic system plus immunosuppression leads to the worst damage and subsequent death.

The positivity of parasitological evaluations (HC and PCR) was also higher in immunosuppressed animals (Table 1), regardless inoculation route, while the serology was always negative, as expected due to immunosuppression. However, in animals non immunosuppressed and parasitologically negative (subpatent parasitemia) and HC and PCR negative, the serology was positive (Table 2), which indicates the presence of low infection and subpatent parasitemia. It is important to highlight that in non-immunosuppressed animals the rates of HC and PCR positive were higher at 30 d.p.i., once more confirming the late pattern of parasitemia of the Colombian *T. cruzi* strain in Swiss mice [29].

In the histopathological evaluation of animals ip infected, immunosuppressed, or non-immunosuppressed, signs of infection in the meninges or cortex and hippocampus were not observed at 15th and 30th d.p.i. (not shown), suggesting that the parasites failed to cross the blood-brain barrier (BBB) (Table 3). Other study [37] observed that only 5% of Swiss mice infected via ip with 20,000 blood trypomastigotes of the CFL strain developed a cerebral parasitism. Increased BBB permeability occurs when factors derived from pathogens (e.g., cysteine protease) are recognized by T lymphocytes, which leads to the production of cytokines into SNC, thereby stimulating the brain endothelial cells to produce adhesion molecules that favor cell migration. Such cytokines stimulate astrocytes to produce chemotactic cytokines that increase the permeability of the BBB [38,39,40,41]. On the histopathological evaluation of the brain in non-immunosuppressed animals icv infected not treated (INT icv) evaluated at 15th d.p.i. the inflammation was focal with consequent changes in the parenchyma and meninges (Figure 3 and Figure 4). The presence of the parasite inside the CNS was associated with vascular, inflammatory response and neuronal degeneration, similar to findings of cerebral functional microvascular alterations described, in Swiss Webster mice infected with *T. cruzi* Y strain [42].

Our results also are similar to the study [37] that resulted in 100% of parasitism of the brains (macrophages, astrocytes and axons) of mice infected via intracerebral. The absence of significant changes in the brains of non-immunosuppressed animals evaluated at 30th d.p.i. has been demonstrated in other studies [17,43]. There was a regression of the brain damage along with the evolution of the disease, favoring the lack of anatomic pathological data in patients in the later phase of acute infection and especially in the chronic phase of CD [44]. With the intervention of immunosuppression, we found increased tissue parasitism and damage of the brains in mice. At 15 d.p.i. the animals icv infected presented lesions with increased cellularity, discrete signs of cellular degeneration and frequent presence of parasites nests (Figure 3 and Figure 4A–F). We also observed parasitism in hippocampal cells. Our findings corroborate the study [19] that using C3H/He mice infected with 100 blood trypomastigotes of the Colombian strain, observed several CNS areas affected, including the hippocampus, a region of the brain that is part of the limbic system, which regulates emotions and is associated to memory, depression, and seizures [45].

Both groups (carbachol + icv) evaluated at 15 d.p.i (immunosuppressed and non-immunosuppressed) presented focal inflammatory lesions in the cortex, more intense in the non-immunosuppressed group (Figure 3 and Figure 4), possibly due to the low parasitemia and the beginning of the development of immune response against the parasite. No parasitism was observed in the brain of mice non immunosuppressed, what could be explained by the low parasitemia found. The hypothalamic lesion observed in the carbachol + icv immunosuppressed group was less intense than in the group only infected via icv (Figure 3E,F and Figure 4) and the atropine + icv group (Figure 3I,J and Figure 4). These results point to the protective role of carbachol in the *T. cruzi* induced brain lesions. Cellular invasion by *T. cruzi* trypomastigotes from cell culture, as used in this study, is not dependent on the activation of mammalian target of rapamycin (mTOR) and may involve autophagy/endocytosis by the host cell for parasite internalization. To this end, molecules secreted and expressed on the surface of the parasite have been implicated in its internalization, such as cruzipain, sialic acid and glycoprotein of different kDa [46]. Moreover, *T. cruzi* has a membrane trans-sialidase that activates in the cytosol the tyrosine kinase receptor (TrKA) primarily by nerve growth factor (NGF), promoting autophosphorylation and PI3K/Akt signaling, an anti-apoptotic molecule [28,47]. TrKA are expressed in all cholinergic neurons, in the brain and peripheral nervous system, in most sympathetic and sensory nociceptive neurons, and in more than 75% of enteric cholinergic system [48]. In addition, a neurotrophin-3 receptor (TrkC) is activated by the parasite and mediates cell invasion in the host [49].

Therefore, we hypothesize that the host cells of the carbachol + icv group, when exposed to the agonist of the mAChR (carbachol), promotes activation of membrane PLC (phospholipase C) and increases of IP3 (inositol 1,4,5-triphosphate) and consequently of Ca^2+^, mainly via mAChR M1, initially facilitating the lysosomal exocytosis and the cell invasion by *T. cruzi*. However, this hypothesis is not supported by the tests here employed. This may respond for the occurrence of higher parasitemia observed after the 12th d.p.i. However, after the initial facilitation of the cellular invasion by the parasite, possibly a competition between the pathways, involving the PLC recruited by the parasite and the mAChR activation pathway by the carbachol activated G protein Gq/11 occurred, with consequent reduction of the parasitemia observed after the 18th d.p.i. In addition to the aforementioned factors, the activation of mAChR M2 and M4 by carbachol, inhibits the production of cAMP via G protein (Gi), an important molecule for the potentiation of lysosomal exocytosis regulated by Ca^2+^ and parasite internalization. Therefore, the probable combination of the latter possibilities hinders and/or prevents the increase of the cellular infection by *T. cruzi* with consequent reduction of the parasitemia and preservation of the brain structures in these animals. Another aspect is that, because carbachol is a primary muscarinic agonist that also has an affinity for nicotinic receptors, both effects could be acting simultaneously at the beginning of the infection.

In non-immunosuppressed animals of the atropine + icv group evaluated 15 d.p.i, amastigotes were observed in the middle of the inflammatory infiltrate (Figure 4K–L). These findings corroborate with [18] publication that revealed edema, increased perivascular spaces, focal meningoencephalitis, and perivascular and parenchymal mononuclear infiltrates irregularly distributed throughout the CNS during the acute phase. This study also showed evidences for incomplete BBB areas, such as the choroid plexus and hippocampus, with intense inflammation and inflammatory infiltrates during the acute infection. At 30 d.p.i., only discrete, non-specific changes were noticed without parasitism (Figure 5).

With the intervention of immunosuppression, animals of the atropine + icv group evaluated 15th d.p.i presented all areas of the brain parasitized, showing important degenerative changes of the parenchyma such as necrosis in the periventricular area, areas of cortical degeneration, and edema (Figure 3 and Figure 4 and Table 3). There was significant parasitism in the parenchyma and meninges with the presence of more intact amastigotes nests. In various areas, parasites and inflammatory infiltrate were associated with degenerative neurons and cell death (Figure 4I,J). It seems that with the mAChR blocked by atropine, the intracellular signaling that triggers apoptosis was not blocked by the parasite and the consequence is strong damage in the brain. The hippocampus and cortex were extensively damaged and these areas have a high presence of mAChR [50]. The parasitism, inflammation and vascular reactivity of the atropine + icv group were much more intense than in animals only icv infected and in animals that had cholinergic receptors activated (carbachol + icv). These important histopathological changes and the high parasitism found in the immunosuppressed animals of the atropine + icv group (treated with antagonist muscarinic cholinergic receptor), would explain the high mortality rate observed at 15 days of evaluation, exactly when the parasitemia began to increase.

Regarding the motor impairment and balance measured by the Rotarod test, the activation of the cholinergic pathway (carbachol + icv) immunosuppressed induced a better locomotor state, since the mice remained longer time on the rotating stem when compared to atropine + icv and INT icv groups immunosuppressed (Figure 6A,B). The blockade of the cholinergic pathway in the infection process by the Colombian strain compromised the locomotor status of the animals, since those of the atropine + icv group remained in the rotating stem for much less time than mice only infected via icv (Figure 6A,B). In the animals infected and treated with atropine via icv (atropine + icv), the immunosuppression favored more yet the motor deficits, which may be explained by the extensive damage of the cortex and hippocampus in the immunosuppressed animals of this group. In non-immunosuppressed groups infected with *T. cruzi* evaluated at 15 d.p.i., mice inoculated via icv and not treated (INT icv) presented motor and balance impairment, remaining less time on the stem of the Rotarod apparatus when compared to animals INT ip and the controls carbachol icv and PBS icv groups, which remained for almost 120 s in equilibrium (Figure 6B). The locomotor activity of the animals remained normal when inoculated with PBS via icv (PBS icv) and with *T. cruzi* via ip. (INT ip). Thus, the inoculation of *T. cruzi* via icv seems to promote damage in brain areas important for motor activity, which would explain motor impairment observed in these animals.

It is well known that acetylcholine is one of the major neurotransmitters involved in higher brain functions, including cognitive processes such as learning, memory, attention, and extrapyramidal locomotor activity [51]. The study [19] in C3H/He mice, considered more susceptible to the development of CNS inflammation in infections with Colombian *T. cruzi* strain, locomotor and exploratory changes were not observed. However, these changes were present in the C57BL/6 mice, more resistant to CNS inflammation, when evaluated by the open field test used for locomotor evaluation [52]. Using the same test used in our study, these authors verified that the animals exhibited at acute phase a significant decrease in locomotor/exploratory activity compared to controls not infected.

As a conclusion, we characterized an intraventricular model of *T. cruzi* CNS infection with Colombian *T. cruzi* strain. Our model induced parenchymal brain parasitism and damage and allowed the investigation of the cholinergic receptors’ participation in the infectious process, lesions, and behavioral changes. The blockade of mAChR with atropine increased the parasitemia, parasitism and damage compared to its activation with carbachol. Cholinergic receptor blockade also increased impairment of coordination vs. receptor activation.

## 4. Materials and Methods

### 4.1. Animals and Ethic Aspects

Four hundred male Swiss mice, 28 to 30 days old, from Centro de Ciência Animal (CCA), Universidade Federal de Ouro Preto (UFOP) were maintained following the guidelines established by the Conselho Nacional de Controle de Experimentação Animal (CONCEA) according to the international guidelines. Animals were kept in a conventional room at 20 to 24 °C, 12–12 h light-dark cycle, and receiving filtered water and balanced commercial feed “ad libitum”. All procedures were approved by the institutional Comitê de Ética em Experimentação Animal (CEUA-UFOP), MG, Brazil, protocol number 2017-36.

### 4.2. Inoculum

For infection of the animals it was used the original Colombian *T. cruzi* strain [53], belonging to DTU I or genotype [54], because it infects cells of the CNS [17,43,55]. Parasites preserved in liquid Nitrogen was cultivated in LIT media at 28 °C and used for Vero cells infection. Trypomastigotes obtained on the 9th day of culture in these cells at 37 °C in 5% CO_2_ were used for mice inoculation.

### 4.3. Infection and Treatment of the Animals

Groups of mice, immunosuppressed or non-immunosuppressed were infected with 2000 trypomastigotes of the original Colombian strain, via intracerebroventricular (icv) and intraperitoneal (ip). The animals were previously treated with a single dose of the agonist carbachol (Sigma, São Paulo, SP, Brazil) (0.005 mL/animal of 3 µM solution) or the antagonist atropine (Sigma, São Paulo, SP, Brazil) (0.005 mL/animal of 20 µM) of the cholinergic receptors for investigation of the role of the muscarinic cholinergic receptors in *T. cruzi* infection evolution. For immunosuppression, the animals were treated with cyclophosphamide monohydrate (2 mg/day, via ip) from third to sixth d.p.i. (first cycle) according to [56] protocol modified. After three days of interval, only one cycle (10th to 13th d.p.i) was repeated to avoid precocious mortality of the animals and allowing parasitological, histopathological, and behavioral evaluations.

The inoculation was performed according to [57] with modifications, using a contender apparatus (ScienLabor, Ribeirão Preto, SP, Brazil) adapted for icv injection (Figure S1). Trypomastigotes derived from Vero cells were used because it was not possible and convenient to inject blood trypomastigotes by icv route where the space is limited. Moreover, blood cells could induce local lesions.

### 4.4. Experimental Groups

Two hundred forty mice, divided into experimental groups with 10 animals each, were evaluated according to the experimental design 1 (Figure S2A). One hundred and twenty mice were immunosuppressed and 120 were non immunosuppressed. Half of each subgroup (60 mice) were evaluated 15 d.p.i. and a half (60 mice) were evaluated 30 d.p.i. The evaluation at the 15th d.p.i. was performed due to the precocious mortality observed in the immunosuppressed animals.

#### 4.4.1. Mice Immunosuppressed Evaluated at 15 d.p.i. (60 Mice)

Carbachol + icv: Previously treated via icv with carbachol (agonist) and infected via icv; atropine + icv: Previously treated via icv with atropine (antagonist) and infected via icv; INT icv: Infected via icv not treated; INT ip: Infected via ip not treated.

#### 4.4.2. Mice Immunosuppressed Evaluated at 30 d.p.i. (60 Mice)

Carbachol + icv: Previously treated via icv with carbachol (agonist) and infected via icv; atropine + icv: Previously treated via icv with atropine (antagonist) and infected via icv; INT icv: Infected via icv not treated; INT ip: Infected via ip not treated.

#### 4.4.3. Mice Non Immunosuppressed Evaluated at 15 d.p.i. (60 Mice)

Carbachol + icv: Previously treated via icv with carbachol (agonist) and infected via icv; atropine + icv: Previously treated via icv with atropine (antagonist) and infected via icv; INT icv: Infected via icv not treated; INT ip: Infected via ip not treated; PBS icv: Injected with PBS via icv: NC: Normal control.

#### 4.4.4. Mice Non Immunosuppressed Evaluated at 30 d.p.i. (60 Mice)

Carbachol + icv: Previously treated via icv with carbachol (agonist) and infected via icv; atropine + icv: Previously treated via icv with atropine (antagonist) and infected via icv; INT icv: Infected not treated via icv; INT ip: Infected not treated via ip; PBS icv: Injected with PBS via icv; NC: Normal control.

### 4.5. Evaluation of the Animals

#### 4.5.1. The Curve of Parasitemia (PAR)

Animals were daily evaluated by fresh blood examination (FBE) according to [58], from 6th d.p.i. up consistent negativity of the parasitemia for at least five consecutive days. PAR was plotted using the daily media counting of trypomastigotes/0.01 mL of blood.

#### 4.5.2. Hemoculture (HC)

HC was performed following [59]. All tubes were examined at 30, 60, 90, and 120 days later.

#### 4.5.3. Molecular Parasitological Method (PCR)

The PCR (Polymerase Chain Reaction) used 0.2 mL of blood obtained from the orbital plexus of each animal before necropsy (15 and 30 d.p.i.). Aliquot of 0.2 mL of the lysate in Guanidine/EDTA was subjected to DNA extraction (WizardTM Genomic DNA Purification kit—Promega, Madison, USA), following manufacturer’s recommendations. PCR reaction was processed according to [60] as standardized in our laboratory using the primers: #121 (AAATAATGTACGGGTGAGATGCATGA) and #122 (GGTTCGATTGGGGTTGGTGTAATATA) (DTTP-Sigma, St. Louis, MO, USA) Invitrogen, São Paulo, SP, Brazil) and Taq Platinum DNA polymerase (Invitrogen, São Paulo, SP, Brasil). Negative, positive and reagents controls were used. Negative samples were submitted to TNF-α mice gene amplification as internal control. The amplified DNA was visualized by electrophoresis in an electronic gel.

#### 4.5.4. Serological Method (ELISA-Enzyme Linked Immunosorbent Assay)

ELISA was carried out in serum samples diluted 1:80 from surviving animals collected before necropsy (15 and 30 d.p.i) according to [61] modified and standardized as [62]. Bruit alkaline antigen [63] peroxidase-labeled anti-mouse IgG conjugate (SIGMA, St. Louis, MO, USA) and the substrate solution of ortho-phenylene diamine (OPD) were used. The reaction was read in a spectrophotometer (Bio-Rad, Berkeley, USA, Model 680—microplate manager 5.2.1) and using a filter of 490 nm. Samples containing absorbance values above the cut-off (mean absorbance of 10 standard negative sera + 2X the standard deviation) were considered reactive, and those with absorbance values below the cut-off were considered negative. All samples were tested in triplicate.

#### 4.5.5. Mortality

The mortality of the animals was daily evaluated until necropsy (15th and 30th d.p.i) and was expressed as a cumulative percentage.

#### 4.5.6. Histopathological Evaluations

The surviving animals were euthanized at 15 and 30 d.p.i by cervical dislocation technique after anesthesia with ketamine and xilaxin (5 mg and 0.5 mg/25 g weight), ip. Under necropsy, the brains were collected and preserved in 10% neutral-buffered formalin. Coronal brain slices were routinely processed, paraffin-embedded, and stained by Hematoxylin-Eosin (HE) for microscopic evaluation of inflammatory processes and the presence of parasites. The histopathological findings were documented in frontal lobe sections near the longitudinal cleft, hippocampus, choroid plexus and peri-ventricular areas to allow comparisons between groups and periods of infection. Images were obtained in light microscopy and captured at 1392 × 1040 pixel resolution. They were transferred via a color video camera—cool-SNAP-PRO Color (Color Cybernetics, Bethesda, MD, USA) for a video system coupled to a computer using the program Image-Pro Express version 4.0 for Windows (Cybernetics media, Bethesda, MD, USA). The interpretation by one pathologist was blinded. Semi-quantitative histopathological score: Using a bright field microscope, under the 20X objective, the 15 d.p.i. brain sections H&E-stained were analyzed for the presence of edema, hyperemia, neurodegenerative changes, inflammatory infiltrate, extracellular and intracellular parasitism. Brains of four representative animals of each group (n = 4/group) were attributed a score in each aspect evaluated. The score was plotted in a scale score based on the intensity of the changes: 0, absence, +, discrete changes, ++, moderate changes, +++, intense changes, very intense. (F) focal and (D) diffuse lesions were characterized (Table 3, Figure 3).

### 4.6. Behavioral Studies

Additionally, 80 mice were infected as previously via icv (60 mice) and ip (20 mice), evaluated at 15 d.p.i and then submitted to behavioral test in parallel to other 80 non-infected animals, divided into two subgroups immunosuppressed (40 mice) and non-immunosuppressed (40 mice). The total number of mice (160) submitted to behavioral test were subdivided into 16 subgroups, with 10 animals each, as shown in the experimental design 2 (Figure S2B).

#### 4.6.1. Groups Infected and Immunosuppressed (40 Mice)

Carbachol + icv: Previously treated via icv with carbachol (agonist) and infected via icv; atropine + icv: Previously treated via icv with atropine (antagonist) and infected via icv; INT icv: Infected not treated via icv; INT ip: Infected not treated via ip.

#### 4.6.2. Groups Infected and Non Immunosuppressed (40 Mice)

Carbachol + icv: Previously treated via icv with carbachol and infected via icv; (6) atropine + icv: Previously treated via icv with atropine and infected via icv; INT icv: Infected not treated via icv; INT ip: Infected not treated via ip.

#### 4.6.3. Groups Not Infected and Immunosuppressed (40 Mice)

Carbachol icv: Treated with carbachol via icv; atropine icv: Treated with atropine via icv; PBS icv: Injected with PBS via icv; NC: Normal control.

#### 4.6.4. Groups Not Infected and Non Immunosuppressed (40 Mice)

Carbachol icv: Treated with carbachol via icv: atropine icv: Treated with atropine via icv; PBS icv: Injected with PBS via icv; NC: Normal control.

### 4.7. Assessment of Motor Coordination and Equilibrium

Experiments for evaluation of motor coordination balance of the animals were performed on the Rotarod apparatus (EFF412, Insight, Ribeirão Preto, SP, Brazil) as described in [64] and carried out with some modifications. The Rotarod is a rotating cylinder with a radius of 2.7 cm and a height of 40 cm. The device has four bays with 3 cm of space for each mouse. Four animals were evaluated in parallel. Animals that remain for a longer time in the rotating system of the device were considered better in motor coordination and equilibrium. The performance of Rotarod was continuously measured on a rotating rod since the mice should move forward to avoid falling. The animals were initially exposed to a pre-training for two consecutive days at four different speeds (16, 24, 28, and 32 rpm) for 3 min each, following the protocol of [65] modified. Animals that remained up 5 s were excluded according to [66]. On the 30th day, the animals were again placed on the rod three times in the accelerated mode when the residence time on the rotating rod in sec was recorded for up two minutes as [67].

### 4.8. Statistical Analysis

One-way ANOVA or Mann–Whitney U tests were used to compare the curve of parasitemia between groups. The mortality of the different experimental groups was analyzed by the Log-rank (Mantel–Cox) test. One-way ANOVA with SNK post-hoc was used to compare the semi-quantitative histopathological data between the experimental groups. The results of the behavioral tests were analyzed by the parametric statistics methods. The percentages of latency before to fall from the Rotarod apparatus were subjected to analysis of variance (ANOVA) followed by the Newman–Keuls test and were presented as the significant and standard error of the mean (SEM) using the program statistic GraphPad Prism 7. Statistical differences between groups were considered significant when *p* < 0.05.

## Figures and Tables

**Table 1:** Positivity of the laboratorial examinations and mortality in immunosuppressed Swiss mice infected via intracerebroventricular and intraperitoneal with 2000 trypomastigotes of the Colombian *Trypanosoma cruzi* of Vero cells previously treated with carbachol (agonist cholinergic) and atropine (antagonist cholinergic) evaluated for 15 and 30 days post-infection.

Infection	Period of Evaluation	Experimental Groups	Positivity of the Laboratorial Tests (%)			Mortality (%)
			HC	PCR	ELISA	
Acute Phase Colombian *T. cruzi* strain Mice Immunosuppressed	15 d.p.i.	INT ip	100% (6/6)	100% (6/6)	0% (0/6)	0% ^@^ (0/10)
		INT icv	90% (9/10)	90% (9/10)	0% (0/10)	40% ^@^ (4/10)
		carbachol + icv	100% (7/7)	100% (7/7)	0% (0/7)	30% (3/10)
		atropine + icv	100% (4/4)	100% (4/4)	0% (0/4)	60% (6/10)
	30 d.p.i.	INT ip	100% (5/5)	100% (5/5)	0% (0/5)	50% (5/10)
		INT icv	100% (9/9)	100% 9/9)	0% (0/9)	10% ^$,&^ (1/10)
		carbachol + icv	-	-	-	100% ^$^ (10/10)
		atropine + icv	-	-	-	100% ^&^ (10/10)

d.p.i.: days post infection; INT ip: Infected via intraperitoneal and not treated; INT icv: Infected via intracerebroventricular and not treated; carbachol + icv: Group previously treated with carbachol and infected via intracerebroventricular; atropine + icv: Group previously treated with atropine and infected via intracerebroventricular; HC: Hemoculture; PCR: Polymerase Chain Reaction; ELISA: Enzyme-Linked-Immuno-Sorbent-Assay serological test. ^@,&,$^ Different symbols indicate differences statistically significant between rate of mortality (Log-rank (Mantel-Cox) test, *p* < 0.05).

**Table 2:** Positivity of the laboratorial examinations and mortality in non immunosuppressed Swiss mice infected via intracerebroventricular and intraperitoneal with 2000 trypomastigotes of the Colombian *Trypanosoma cruzi* of Vero cells treated with carbachol and atropine evaluated 15 and 30 days post-infection.

Infection	Period of Evaluation	Experimental Groups	Positivity of the Laboratorial Tests (%)			Mortality (%)
			HC	PCR	ELISA	
Acute Phase Colombian *T. cruzi* strain	15 d.p.i.	INT ip	50% (5/10)	40% (4/10)	100% (10/10)	0% (0/10)
		INT icv	66.66% (6/9)	22.22% (2/9)	100% (9/9)	10% (10/10)
		carbachol + icv	50% (4/8)	50% (4/8)	100% (8/8)	20% (2/10)
		atropine + icv	75% (6/8)	37.5% (3/8)	100% (8/8)	20% (2/10)
	30 d.p.i.	INT ip	60% (6/10)	80% (8/10)	100% (10/10)	0% (0/10)
		INT icv	88.88% (8/9)	88.88% (8/9)	100% (9/9)	10% (1/10)
		carbachol + icv	75% (6/8)	87.5% (7/8)	100% (8/8)	20% (2/10)
		atropine + icv	100% (8/8)	100% (8/8)	100% (8/8)	20% (2/10)

d.p.i.: days post-infection; INP ip: Infected via intraperitoneal and not treated; INT icv: Infected via intracerebroventricular and not treated carbachol + icv: Group previously treated with carbachol and infected via intracerebroventricular; atropine + icv: Group previously treated with atropine and infected via intracerebroventricular; HC: Hemoculture; PCR: Polymerase Chain Reaction; ELISA: Enzyme-Linked-Immuno-Sorbent-Assay serological test.

**Table 3:** Semi-quantitative histopathological score in the brain of Swiss mice Swiss mice infected via intracerebroventricular and intraperitoneal with 2000 trypomastigotes of the Colombian *Trypanosoma cruzi* of Vero cells treated with carbachol (agonist cholinergic) and atropine (antagonist cholinergic), immunosuppressed and non immunosuppressed evaluated 15 days post infection.

	Experimental Groups	Edema	Hyperemia	Neurodegenerative Changes	Inflammatory Infiltrate	Extracellular Parasitismo	Intracellular Parasitismo	Most Affected Locations
Mice Immunosuppressed	INT icv	+	0	+	+++ (F)	+	+++	Meninge and parenchymal region
	Carbachol + icv	++	0	++	++ (F)	0	++	Meninge, hippocampus and callosum
	Atropine + icv	++	+++	+++	+++ (F)	0	++++	Meninge and parenchyma
	INT ip	0	0	0	0	0	0	0
	PBS icv	+	+	0	++	0	0	Meninge
	NC	0	0	0	0	0	0	0
Mice Non Immunosuppressed	INT icv	+	0	++	++	0	+ (F)	Meninge and parenchymal region
	Carbachol + icv	++ (F)	+	++ (F)	++ (F)	0	+	Meninge and cortex
	atropine + icv	+	+	+ (F)	+ (F)	+	++	Cortex
	INTip	0	0	0	0	0	0	0
	PBS icv	0	0	0	+	0	0	Meninge
	NC	0	0	0	0	0	0	0

Score—0: absence; +: Discrete; ++ Moderate; +++: Intense; Very intense: ++++; (F): Focal; (D) Difuse; INT icv: Infected and not treated inoculated via icv; INT ip: Infected and not treated inoculated via ip; carbachol + icv infection: Infected and previously treated with carbachol via icv; atropine + icv infection: Infected and previously treated with atropine via icv; carbachol icv: Treated with carbachol via icv; atropine icv: Treated with atropine via icv; PBS icv: Inoculated with PBS via icv; NC: Normal control.

## Data Availability

Not Applicable.

## Supplementary Materials

The following are available online at https://www.mdpi.com/2076-0817/10/2/121/s1, Figure S1: Contender apparatus for mice and local of inoculation of the trypomastigotes of Vero cells. (A) Contender apparatus (ScienLabor, São Paulo, Brazil) adapted for mice intracerebroventricular (icv) injection. (B) Design showing the local (black point) of intracerebroventricular inoculation in mice. Figure S2: (A) Experimental design 1: Infection of Swiss mice with Colombian *Trypanosoma cruzi* strain and experimental groups. (B) Experimental design 2: Behavioral tests. N: Number of mice, icv: Intracerebroventricular, ip: Intraperitoneal, d.p.i: days post-infection, INT icv: Infected via icv and not treated with carbachol or atropine, INT ip: Infected via ip and not treated, carbachol+icv: previously treated with carbachol (agonist) via icv and infected via icv, atropine+icv: previously treated with atropine (antagonist) via icv and infected via icv, NC: Normal Control, PBS icv: Inoculated with Phosphate Buffer Solution via icv, PAR: Parasitemia curve, carbachol icv: treated with carbachol via icv, atropine icv: treated with atropine via icv.
