# Supplementary material for: Participation of Central Muscarinic Receptors on the Nervous Form of Chagas Disease in Mice Infected via Intracerebroventricular with Colombian Trypanosoma cruzi Strain
Source: Pathogens. 2021 Jan 25;10(2):121. doi: 10.3390/pathogens10020121 (PMC7922850; doi:10.3390/pathogens10020121)
Supplement: Supplementary file 1 [file pathogens-10-00121-s001.zip › supplmentary/Figure S1.docx]

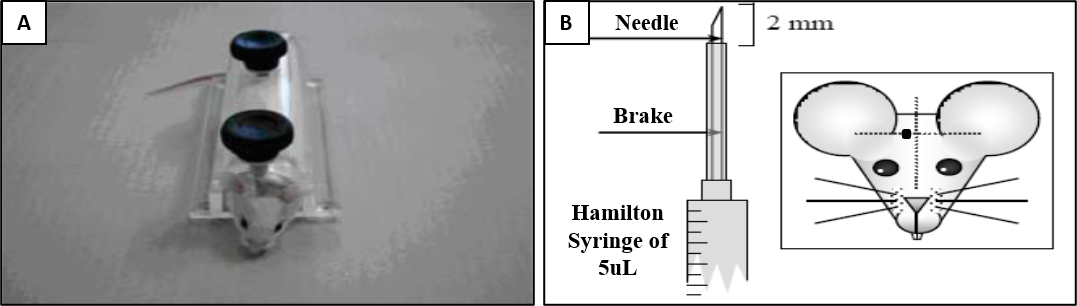


**Figure S1:** Contender apparatus for mice and local of inoculation of the trypomastigotes of Vero cells. **A** - Contender apparatus (ScienLabor, São Paulo, BRA) adapted for mice intracerebroventricular (icv) injection. **B** - Design showing the local (black point) of intracerebroventricular inoculation in mice.
