# Supplementary material for: Participation of Central Muscarinic Receptors on the Nervous Form of Chagas Disease in Mice Infected via Intracerebroventricular with Colombian Trypanosoma cruzi Strain
Source: Pathogens. 2021 Jan 25;10(2):121. doi: 10.3390/pathogens10020121 (PMC7922850; doi:10.3390/pathogens10020121)
Supplement: Supplementary file 1 [file pathogens-10-00121-s001.zip › supplmentary/Figure S2.docx]

**
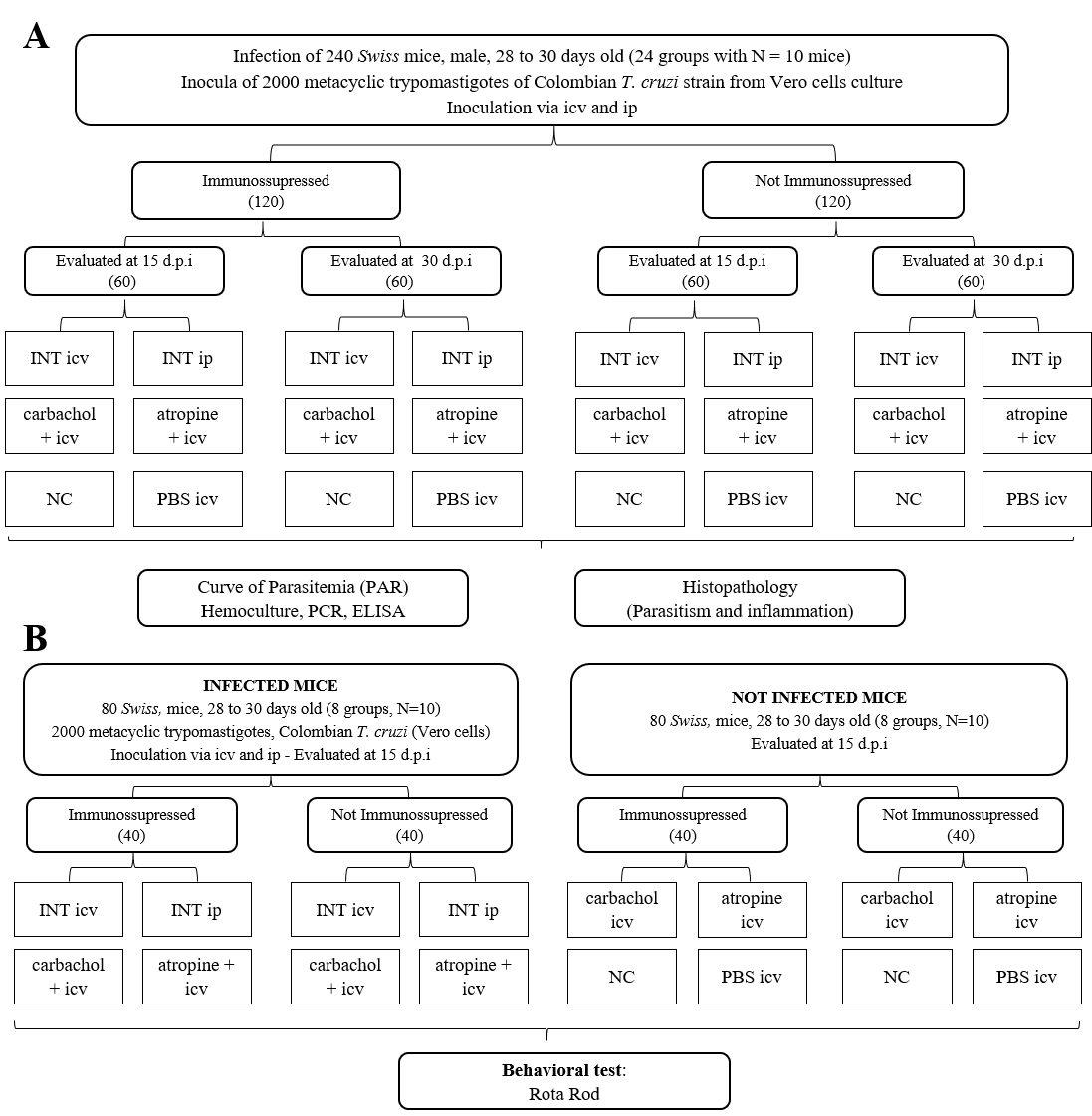
**

**Figure S2:** A: Experimental design 1: Infection of Swiss mice with Colombian *Trypanosoma cruzi* strain and experimental groups. B: Experimental design 2: Behavioral tests.

N: Number of mice, icv: Intracerebroventricular, ip: Intraperitoneal, d.p.i: days post-infection, INT icv: Infected via icv and not treated with carbachol or atropine, INT ip: Infected via ip and not treated, carbachol+icv: previously treated with carbachol (agonist) via icv and infected via icv, atropine+icv: previously treated with atropine (antagonist) via icv and infected via icv, NC: Normal Control, PBS icv: Inoculated with Phosphate Buffer Solution via icv, PAR: Parasitemia curve, carbachol icv: treated with carbachol via icv, atropine icv: treated with atropine via icv.
